# Electrospinning Synthesis of Carbon-Supported Pt_3_Mn Intermetallic Nanocrystals and Electrocatalytic Performance towards Oxygen Reduction Reaction

**DOI:** 10.3390/nano10091893

**Published:** 2020-09-22

**Authors:** Lechao Peng, Lan Zhou, Wenjun Kang, Rui Li, Konggang Qu, Lei Wang, Haibo Li

**Affiliations:** Shandong Provincial Key Laboratory of Chemical Energy Storage and Novel Cell Technology, School of Chemistry and Chemical Engineering, Liaocheng University, Liaocheng 252059, China; lechaopeng@163.com (L.P.); lan_zhou_z@163.com (L.Z.); kangwenjun@lcu.edu.cn (W.K.); lirui@lcu.edu.cn (R.L.); qukonggang@lcu.edu.cn (K.Q.); wanglei@lcu.edu.cn (L.W.)

**Keywords:** electrospinning, Pt_3_Mn intermetallic, oxygen reduction reaction, electrocatalytic performance

## Abstract

To realize the large-scale application of fuel cells, it is still a great challenge to improve the performance and reduce the cost of cathode catalysts towards oxygen reduction reaction (ORR). In this work, carbon-supported ordered Pt_3_Mn intermetallic catalysts were prepared by thermal annealing electrospun polyacrylonitrile nanofibers containing Platinum(II) acetylacetonate/ Manganese(III) acetylacetonate. Compared with its counterparts, the ordered Pt_3_Mn intermetallic obtained at 950 °C exhibits a more positive half-potential and higher kinetic current density during the ORR process. Benefiting from their defined stoichiometry and crystal structure, the Mn atoms in Pt_3_Mn intermetallic can modulate well the geometric and electronic structure of surface Pt atoms, endowing Pt_3_Mn catalyst with an enhanced ORR catalytic activity. Moreover, it also has a better catalytic stability and methanol tolerance than commercial Pt/C catalyst. Our study provides a new strategy to fabricate a highly active and durable Pt_3_Mn intermetallic electrocatalyst towards ORR.

## 1. Introduction

Oxygen reduction reaction (ORR) plays a crucial role in energy storage and conversion devices such as fuel cells and metal–air batteries. Metallic platinum is considered the most efficient ORR electrocatalyst. However, it still suffers from sluggish reaction kinetics in ORR. Moreover, its high cost and low abundance makes it impossible to meet large-scale commercial requirements [1]. To address the above issues, great efforts have been devoted to reducing the consumption of expensive Pt. By finely modulating surface atom arrangement, Pt nanocrystals enclosed by high-index facets display a higher catalytic activity for the equivalent Pt. Covering a thin Pt shell on other metal surfaces also greatly improves the specific activity of the Pt catalyst [2]. Downsizing Pt nanoparticles to clusters and even to single-atom level can maximize Pt utilization efficiency by realizing nearly all Pt atoms at the catalytic interfaces [3].

Constructing PtM catalysts by alloying Pt with 3d transition metals (M) has also attracted tremendous attention and interest, as it not only highly improves the performance of Pt-based catalysts but also efficiently reduces the usage of the Pt component [4]. The enhanced performance is attributed to the synergetic effects of geometric (Pt lattice strain) and electronic (Pt d-band vacancy) structure modulation. Up to now, 3d transition metals including Fe, Co, Ni, Cu, etc. have been widely incorporated into the Pt lattice [5,6,7,8,9], which could efficiently alter the Pt d-band vacancy and lattice strain, providing a suitable binding energy for ORR intermediates [10]. For the majority of PtM alloy catalysts, both Pt and M atoms usually have a random position in the unit cell, i.e., a solid solution structure. During the ORR process, the M component in PtM alloys seriously suffers from the chemical oxidation and etching action, ultimately resulting in a catalytic performance decay [11]. By contrast, the intermetallic PtM alloys with defined stoichiometry and crystal structure combine high activity and durability, serving as an ideal ORR catalyst [12,13,14]. The enhanced stability arises from the strong binding interaction between the two metal elements, which greatly reduces the mobility of M in the PtM intermetallics [15,16,17].

Nowadays, most studies for Pt-based intermetallics focus on the PtFe, PtCo, and PtNi alloys, but little attention is paid to PtMn, even its disordered phase [18,19,20,21,22]. Recently, it was found that the PtMn intermetallic can also be applied as an efficient ORR catalyst. For example, B. Murray et al. report that ordered-phase PtMn cubic nanocrystals are much more active for ORR than the pure Pt black and ETEK Pt catalysts [23]. Due to the low standard electrode potential of Mn^2+^/Mn (−1.17 V vs. NHE), the solution-phase synthesis approach for PtMn alloy usually needs a relatively high temperature (~200 °C) [24,25,26]. To obtain PtM intermetallics, a two-step synthesis strategy is widely adopted: (i) the co-reduction or impregnation reduction of Pt/M precursors to disordered PtM alloy; (ii) PtM alloy phase transformation from disorder to order through an annealing treatment at high temperature [23,27,28,29]. In actual applications, carbon supports such as Vulcan XC-72 are usually introduced to load PtM catalysts, further improving their electrocatalytic activity [30,31,32].

Herein, we demonstrate a new strategy to prepare carbon-supported Pt_3_Mn intermetallic catalysts. Firstly, polyacrylonitrile (PAN) nanofibers containing Platinum(II) acetylacetonate/ Manganese(III) acetylacetonate (Pt(acac)_2_/Mn(acac)_3_) are fabricated by an electrospinning technique. After annealing in inert atmosphere, Pt_3_Mn intermetallic nanocrystals anchored on carbon nanofibers (PtMn/CNFs) can be directly obtained. In our approach, it is not required to synthesize disordered-phase PtMn precursors in advance, and it also omits the procedure of catalyst loading on carbon supports. The introduction of Mn atoms can well modulate the geometric and electronic structure of Pt catalysts, so the obtained ordered Pt_3_Mn intermetallic displays an improved electrocatalytic performance towards ORR.

## 2. Materials and Methods

### 2.1. Chemicals

Polyacrylonitrile (PAN) was purchased from Sigma-Aldrich Co. (St. Louis, MO, USA). Manganese acetylacetonate (Mn(acac)_3_) and Platinum acetylacetonate (Pt(acac)_2_) were provided by Aladdin Industrial Co. (Shanghai, China). *N*,*N*-dimethylformamide (DMF) was supplied by Tanjin Fuyu Fine Chemical Co., Ltd. (Tianjin, China). All chemicals were used directly without further purification, and all solutions used in electrochemical tests were well prepared with deionized water.

### 2.2. Preparation of Pt(acac)_2_/Mn(acac)_3_/PAN Nanofibers

Following a typical method, 0.09 g Pt(acac)_2_ was added into 12.0 mL DMF and stirred for 0.5 h. Then 1.61 g Mn(acac)_3_ was dissolved into the above solution. Finally, 0.79 g PAN was added, and the mixed solution was further vigorously stirred for 8 h. After leaving this for one night, a homogeneous solution was obtained. In the electrospinning process, the applied voltage was set to 10.8 kV, and the flow rate was controlled by a syringe pump at 6.0 μL min^−1^. An aluminum foil was used to collect the fibers, and its distance to the spinning nozzle (23 gauge) was 15.0 cm.

### 2.3. Preparation of PtMn/CNFs Catalysts

PtMn/CNFs were prepared following our previous work [33]. Briefly, the Pt(acac)_2_/Mn(acac)_3_/PAN nanofibers were firstly stabilized at 230 °C for 3.0 h in air and then further annealed at the target temperature (650~1050 °C) for 1.0 h under N_2_ atmosphere. To remove possible impurities, the carbonized product was fully soaked in 1.0 M HCl for 12 h under magnetic stirring. The final product was well washed with water and ethanol, then it was dried in an oven at 80 °C. For convenience, the PtMn/CNFs were referred to as PtMn-650/CNFs, PtMn-750/CNFs, PtMn-850/CNFs, PtMn-950/CNFs, and PtMn-1050/CNFs on the basis of the annealing temperatures (650, 750, 850, 950, 1050 °C). For comparison, Pt-950/CNFs and Mn-950/CNFs were also carefully prepared by the same method, only in the absence of Mn(acac)_3_ and Pt(acac)_2_, respectively.

### 2.4. Characterization

The crystalline phase was determined by powder X-ray diffraction (XRD) using a Rigaku SmartLab 9 X-ray diffractometer (Tokyo, Japan) with Cu Kα radiation (λ = 1.5418 Å). The morphology was characterized by a scanning electron microscopy (SEM, Zeiss Supra-40, Oberkochen, Germany) and transmission electron microscopy (TEM, FEI Talos F200x, Waltham, MA, USA). Element distribution mapping was carried out using an energy dispersive X-ray detector equipped on the FEI Talos F200x. X-ray photoelectron spectroscopy (XPS) was studied on a Thermo Fisher ESCLAB 250Xi spectrometer (Waltham, MA, USA).

### 2.5. Electrochemical Measurements

The electrochemical tests were carried out on a CHI 832B potentiostat (Shanghai Chenhua Instrument Co., Ltd., Shanghai, China) in a standard three-electrode system. A coiled platinum wire (*d* = 0.5 mm, *L* = 23 cm) and Ag/AgCl (3.0 M KCl) electrode served as the counter and reference electrodes, respectively. The catalyst ink was prepared by dispersing 2 mg catalyst powder in a mixture solution of 1.6 mL water and 0.4 mL isopropanol, followed by sonication for 30 min. The working electrode was fabricated by coating catalyst ink on a glassy carbon rotating disk electrode (*S* = 0.1256 cm^2^), and the Pt loading was kept at ~11 μg cm^−2^. For comparison, a commercial Pt/C (20 wt %, Shanghai Hesen Electric Co., Ltd., Shanghai, China) catalyst was also loaded onto the working electrode with Pt loading of ~15 μg cm^−2^.

## 3. Results and Discussion

### 3.1. Structure and Morphology Characterization

**Figure 1:**
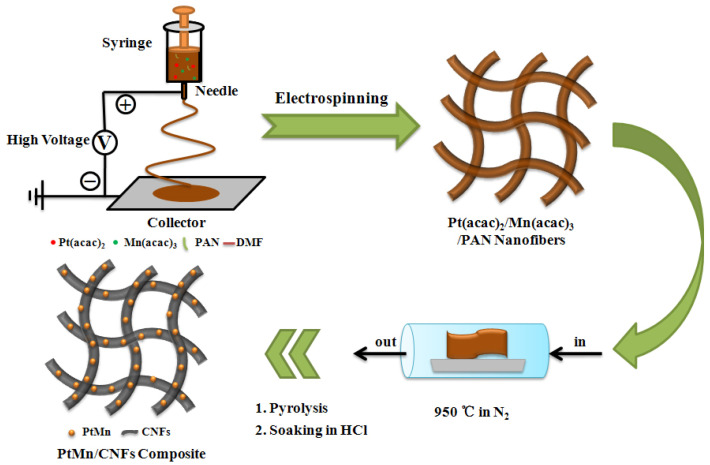
Schematic diagram for the preparation of PtMn/carbon nanofibers (CNFs).

**Figure 2:**
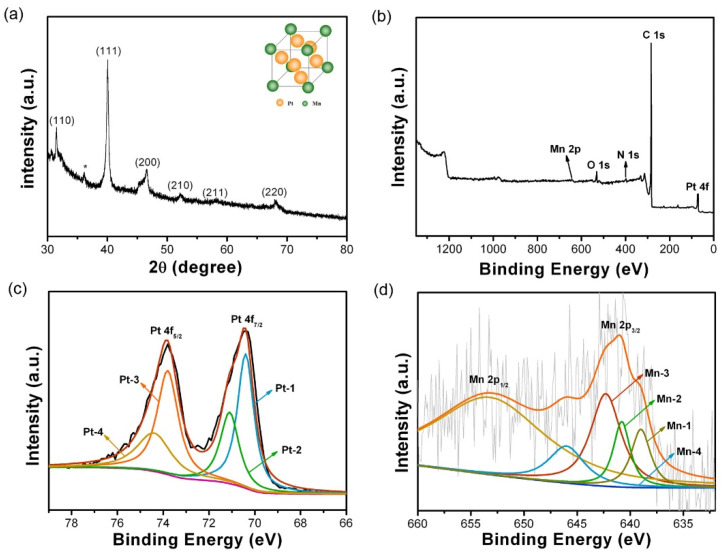
(**a**) XRD pattern and (**b**) XPS survey spectrum of PtMn-950/CNFs. Peak-fitting XPS spectra of (**c**) Pt 4f and (**d**) Mn 2p_3/2_.

Figure 1 shows the schematic illustration for the preparation of PtMn/CNFs, in which electrospinning, pyrolysis, and soaking treatment were involved in sequence. The phase composition was investigated by powder XRD. An L1_2_ phase (AuCu_3_ structure) of Pt_3_Mn (JCPDS: No. 65-3260) was identified in the PtMn-950/CNFs (Figure 2a). As shown in the model (inset of Figure 2a), the Pt atoms occupy the face centers of the unit cell, and the Mn atoms are located at the vertices in the ordered Pt_3_Mn phase [23,29]. Apart from the diffraction peaks of ordered Pt_3_Mn, the peak at ~36.2 ° (marked with *) is likely related to the Mn-rich phase, which was also reported in a previous study [18]. It was found that the annealing temperature had a great influence on the phase composition of PtMn/CNFs (Figure S1). When the temperature was lower than 650 °C, there was no obvious diffraction peak, implying an amorphous phase. A disordered A_1_ phase (JCPDS No. 65-5033) with a face-centered cubic structure was prepared when it rose to 750 °C [29]. Beyond 850 °C, it induced a phase conversion from a disordered A_1_ phase to an ordered L1_2_ phase. Further increasing the temperature to 1050 °C had no effect on the PtMn phase except for the crystallinity. This suggests that the L1_2_ phase with an ordered intermetallic structure is thermodynamically stable at a high temperature.

The XPS technique was applied to check the near-surface element compositions and chemical states. For the PtMn-950/CNFs (Figure 2b), the sharp C1s peak at ~284 eV comes from the carbon nanofibers. Additionally, Pt 4f and Mn 2p peaks are also to be observed, confirming the presence of Pt and Mn elements. The high resolution Pt 4f can be fitted to two pairs of doublets (Figure 2c). The two peaks located at 70.4 and 73.8 eV correspond to the Pt 4f_7/2_ (Pt-1) and Pt 4f_5/2_ (Pt-3) of metallic state Pt^0^, while the other two peaks at 71.1 and 74.4 eV can be assigned to the Pt 4f_7/2_ (Pt-2) and Pt 4f_5/2_ (Pt-4) of Pt^2+^ species. Similarly, the deconvolution of Mn 2p_3/2_ (Figure 2d) also implies the multiple chemical states of Mn: Mn^0^ (639.0 eV, Mn-1), Mn^2+^ (640.8 eV, Mn-2), and Mn^4+^ (624.3 eV, Mn-3) [34]. The Mn-4 at 646.0 eV is the satellite peak of Mn^2+^. It was found that the majority of surface Pt atoms were in a metallic state, while most Mn atoms existed as the oxidation state. A similar phenomenon was also reported in the PtBi intermetallic catalyst [16]. The peak fitting for PtMn-X/CNFs (X = 650, 750, 850, 1050) cases also exhibited similar patterns (Figure S2).

**Figure 3:**
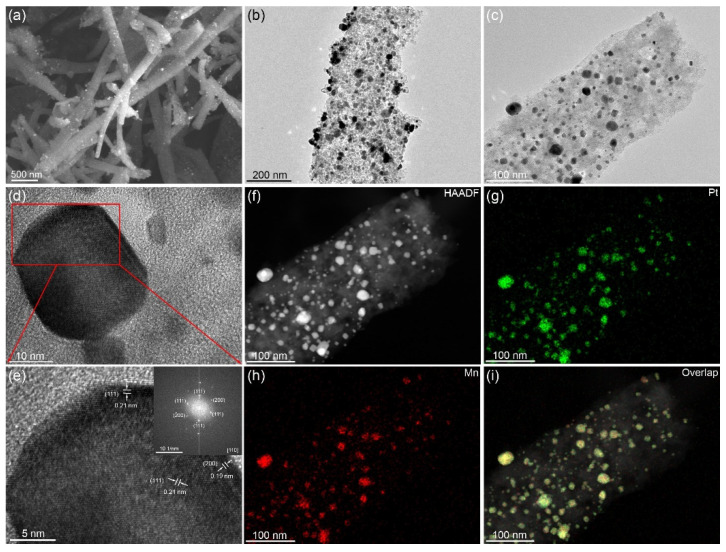
(**a**) SEM and (**b**,**c**) TEM images of PtMn-950/CNFs. (**d**,**e**) HRTEM image of a random PtMn nanoparticle for PtMn-950/CNFs. (**f**–**i**) High-angle annular dark-field scanning transmission electron microscope (HADDF-STEM) image and element (Pt and Mn) mappings for PtMn-950/CNFs.

The microstructure of PtMn-X/CNFs was investigated by SEM and TEM techniques. Figure 3a shows the SEM image of PtMn-950/CNFs, which has a one-dimensional fibrous structure with rough surfaces. The TEM images in Figure 3b,c reveal that small nanoparticles with an average size of ~20 nm are anchored on the fiber surfaces. To investigate the phase of nanoparticles, (HRTEM) technique was applied. The HRTEM images in Figure 3d,e show well-defined lattice spacings of 0.21 and 0.19 nm, corresponding to the (111) and (200) facets, respectively. Compared with the pure Pt (0.23 nm), their values become smaller due to the incorporation of small-sized Mn atoms. Fast Fourier transform (FFT) electron diffraction (inset of Figure 3e) reveals the single-crystalline feature of Pt_3_Mn nanocrystals. A comprehensive analysis of high-angle annular dark-field scanning transmission electron microscope (HADDF-STEM) image and element mapping (Figure 3f‒i) manifests the homogeneous distribution of both Pt (green color) and Mn (red color) elements. Energy dispersive spectrum (EDS) reveals that the Pt content in PtMn-950/CNFs is 13.72 wt % (Figure S3). We also investigated the impact of the annealing temperature on the microstructure of PtMn-X/CNFs (Figure S4). As the annealing temperature increases, the size of the PtMn nanoparticles (NPs) becomes larger, which is caused by the sintering and coarsening of particles at a high temperature [10,14,28]. Moreover, more mesopores appear in the carbon fibers, which can be attributed to the overflow of Pt_3_Mn nanoparticles from carbon materials at a high temperature, leaving rich voids in the carbon fibers.

### 3.2. Electrochemical Performances

**Figure 4:**
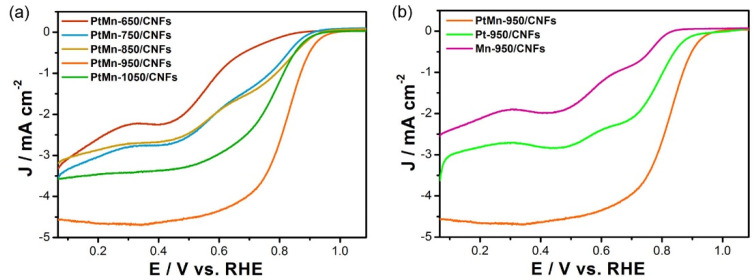
(**a**) Oxygen reduction reaction (ORR) polarization curves of PtMn-X/CNFs (X = 650, 750, 850, 950, and 1050) in O_2_-saturated 0.10 M KOH. (**b**) ORR polarization curves of PtMn-950/CNFs, Pt-950/CNFs, and Mn-950/CNFs in O_2_-saturated 0.10 M KOH (rotation rate: 1600 rpm; sweep rate: 10 mV s^−1^).

The electrocatalytic performances of PtMn-X/CNFs towards ORR were enacted in O_2_-saturated 0.10 M KOH solution using linear sweep voltammetry (LSV). Figure 4a represents the ORR polarization curves of the PtMn-X/CNFs recorded at 1600 rpm. The half-wave potential (*E_1/2_*) of the PtMn-950/CNFs was measured as 0.832 V (vs. RHE), which is far higher than those of the PtMn-650/CNFs (0.569 V), PtMn-750/CNFs (0.692 V), PtMn-850/CNFs (0.751 V), and PtMn-1050/CNFs (0.775 V). To provide a quantitative comparison on intrinsic activity, the kinetic current densities of ORR polarization curves were also calculated by the Koutecky‒Levich (K‒L) equation:

(1) $$\frac{1}{J}=\frac{1}{J_{K}}+\frac{1}{J_{L}}=\frac{1}{J_{K}}+\frac{1}{B\omega^{1/2}}$$

(2) $$B=0.62nFC_{o_{2}}^{*}D_{o_{2}}^{2/3}\upsilon^{-1/6}$$

where *J*, *J_K_*, and *J_L_* are the measured current density, kinetic current density, and the diffusion-limited current density, respectively. *ω* is the electrode rotating rate, *F* represents the Faraday constant (*F* = 96485 C mol^−1^), *n* is the electron transfer number, $C_{O_{2}}^{*}$ is the bulk concentration of O_2_ in 0.10 M KOH (1.2 × 10^−6^ mol cm^−3^), $D_{O_{2}}$ is the diffusion coefficient of O_2_ in 0.10 M KOH (1.9 × 10^−5^ cm^2^ s^−1^), and *ν* is the kinematic viscosity of the electrolyte (0.01 cm^2^ s^−1^).

It was found that PtMn-950/CNFs outperformed other counterparts, and the kinetic current densities at 0.832 V (vs. RHE) followed the order of PtMn-950/CNFs (3.48 mA/cm^2^) > PtMn-1050/CNFs (0.93 mA/cm^2^) > PtMn-850/CNFs (0.91 mA/cm^2^) > PtMn-750/CNFs (0.59 mA/cm^2^) > PtMn-650/CNFs (0.07 mA/cm^2^) (Table S1). Compared with the other counterparts, three structure features could be observed in PtMn-950/CNFs: (i) the formation of ordered Pt_3_Mn intermetallic phase; (ii) a good dispersion of Pt_3_Mn nanocrystals on carbon supports; and (iii) rich pore structures in carbon nanofibers with a facilitated reactant transfer. These merits endowed PtMn-950/CNFs with the highest ORR catalytic activity. Moreover, we also investigated the effect of the catalyst component on the ORR catalytic performance. As shown in Figure 4b, the PtMn-950/CNFs exhibited a much higher ORR catalytic performances than both Pt-950/CNFs and Mn-950/CNFs. This can be attributed to the changes in the electronic and geometric structures of Pt when alloyed with Mn. It has been well proven that the incorporation of transition metal can modulate Pt d-band center, which is highly correlated with the ORR activity. Compared with the Pt-950/CNFs without a Mn component, the binding energies of Pt 4f_7/2_ and Pt 4f_5/2_ in PtMn-950/CNFs were slightly negative-shifted (Figure S5), implying a downshift of d-band center. Two factors were responsible for the above result: (i) the electron transfer from Mn to Pt due to their electronegative difference (Pt: 2.28; Mn: 1.55); and (ii) the compressive strain for their different atomic radii (Pt: 1.38 Å; Mn: 1.24 Å) [17,25,35]. It should be noted that the Mn atoms in intermetallic phase have a much stronger impact on Pt geometric and electronic structures, which make them exhibit a higher activity and stability [36].

**Figure 5:**
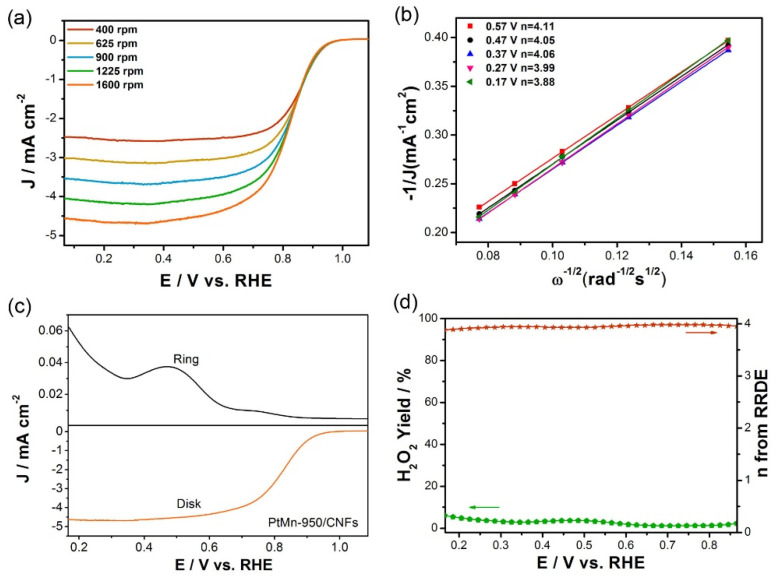
(**a**) ORR polarization curves of PtMn-950/CNFs at different rotation rates in O_2_-saturated 0.10 M KOH. (**b**) Koutecky‒Levich plots of PtMn-950/CNFs at different potentials. (**c**) ORR polarization curves recorded on the RRDE for PtMn-950/CNFs in O_2_-saturated 0.10 M KOH solution at 1600 rpm. (**d**) The calculated H_2_O_2_ yield and electron transfer numbers (*n*) for PtMn-950/CNFs.

To well evaluate the electrocatalytic activity of PtMn-950/CNFs, the polarization curves at different rotation rates (from 400 to 1600 rpm) are recorded (Figure 5a). Then the electron transfer number (*n*) can be obtained from the slope of the K‒L curves by plotting *J*^−1^ vs. *ω*^−1/2^. As shown in Figure 5b, the linearity and parallelism of K–L plots indicate the consistent electron transfer at different potentials and the first-order reaction kinetics with respect to the dissolved O_2_ [37]. The average *n* for PtMn-950/CNFs is calculated to be 4.0 in the potential range from 0.17 V to 0.57 V, implying a high efficient four-electron oxygen reduction process. To gain an insight into the ORR pathway for the PtMn-950/CNFs, rotating ring disk electrode (RRDE) was applied to monitor the H_2_O_2_ yield (Figure 5c). As the potential of Pt ring electrode is set to 0.5 V, the generated H_2_O_2_ from disk electrode can be completely oxidized. According to the following equation, the H_2_O_2_ yield and electron transfer number (*n*) can be determined:

(3) $$H_{2}O_{2}\%=\frac{2I_{R}/N}{I_{D}+I_{R}/N}$$

(4) $$n=\frac{4I_{D}}{I_{D}+I_{R}/N}$$

where *I_D_* is the disk current, *I_R_* is the ring current, and *N* is the current collection efficiency of Pt ring. From Figure 5d, it is found that the H_2_O_2_ yield for the PtMn-950/CNFs in the potential range of 0.17 V~0.87 V is below 6.0 %. The determined electron transfer number (*n*) is 3.80–3.96, being well consistent with the results from the K‒L equation. It further confirms a 4e^−^ catalytic pathway for the PtMn-950/CNFs.

**Figure 6:**
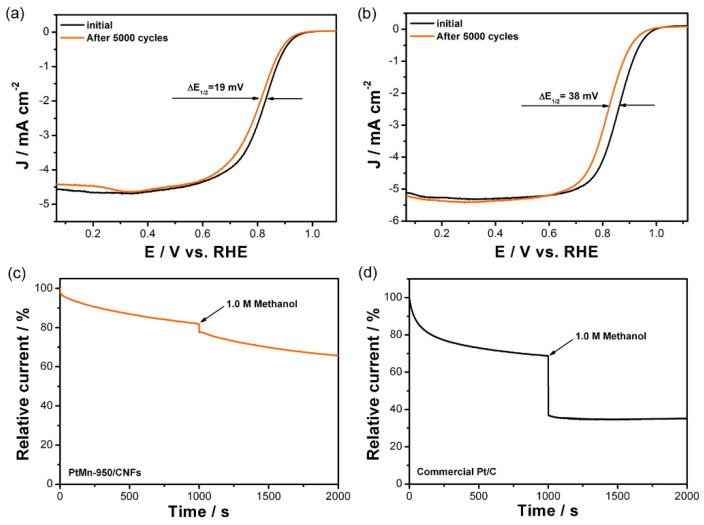
Comparison of ORR polarization curves for (**a**) PtMn-950/CNFs and (**b**) a commercial Pt/C catalyst before and after 5000 cycles in O_2_-saturated 0.10 M KOH solution (rotation rate: 1600 rpm; sweep rate: 10 mV s^−1^). Current-time chronoamperometic responses for (**c**) PtMn-950/CNFs and (**d**) a commercial Pt/C catalyst in O_2_-saturated 0.10 M KOH followed by adding 1.0 M methanol at 1000 s (rotation rate: 1600 rpm; applied potential: 0.82 V).

Accelerated durability tests (ADTs) were conducted to evaluate the catalytic stability of the PtMn-950/CNFs with CV. After 5000 CV cycles from 0.37 to 0.97 V at 100 mV s^−1^, the PtMn-950/CNFs showed a negligible performance loss, and their half-wave potential (*E_1/2_*) negatively shifted by 19 mV, which is lower than that of the commercial Pt/C catalyst (38 mV). Moreover, this still remained a highly efficient four-electron catalytic pathway and a low H_2_O_2_ yield (Figure S6). The catalytic selectivity of the cathode against the fuel oxidation is also highly important in fuel cells, as some fuel molecules, such as CH_3_OH, may penetrate the polymer electrolyte membrane to the cathode, seriously degrading the cell performance [37]. Therefore, a methanol crossover test was performed in O_2_-saturated 0.10 M KOH solution. Figure 6c,d shows the chronoamperometric responses of PtMn-950/CNFs and a commercial Pt/C catalyst at 0.82 V (vs. RHE). Compared with the commercial Pt/C catalyst, the PtMn-950/CNFs kept a relatively stable amperometric response after introducing CH_3_OH molecules at 1000 s, implying a better methanol tolerance feature.

## 4. Conclusions

In summary, carbon-supported ordered Pt_3_Mn intermetallic catalysts were prepared by thermal annealing electrospun Pt(acac)_2_/Mn(acac)_3_/PAN Nanofibers, and their electrocatalytic performances towards ORR were investigated. Due to the defined stoichiometry and crystal structure, the Mn atoms in Pt_3_Mn intermetallic can well modulate the geometric and electronic structure of surface Pt atoms, endowing the Pt_3_Mn catalyst with an enhanced ORR catalytic activity, catalytic stability, and methanol tolerance. Our study provides a new strategy to prepare a highly active and durable Pt_3_Mn intermetallic electrocatalyst, which may find application in fuel cells.

## Supplementary Materials

The following are available online at https://www.mdpi.com/2079-4991/10/9/1893/s1, Figure S1: XRD patterns of PtMn-X/CNFs (X = 650, 750, 850, 1050), Figure S2: XPS survey spectra and peak-fitting Pt 4f and Mn 2p_3/2_ spectra of PtMn-X/CNFs (X = 650, 750, 850, 1050), Figure S3: EDS of PtMn-950/CNFs, Figure S4: TEM images of PtMn-X/CNFs (X = 650, 750, 850, 1050), Figure S5: Pt 4f XPS spectra of PtMn-950/CNFs and Pt-950/CNFs, Figure S6: Electrochemical characterization of PtMn-950/CNFs after ADTs (5000 cycles). Table S1: The calculation of the kinetic current densities at 0.832 V (vs. RHE).
