# Supplementary material for: Electrospinning Synthesis of Carbon-Supported Pt3Mn Intermetallic Nanocrystals and Electrocatalytic Performance towards Oxygen Reduction Reaction
Source: Nanomaterials (Basel). 2020 Sep 22;10(9):1893. doi: 10.3390/nano10091893 (PMC7559926; doi:10.3390/nano10091893)
Supplement: Supplementary file 1 [file nanomaterials-10-01893-s001.pdf]

Supporting Information

Electrospinning Synthesis of Carbon-Supported Pt_3Mn Intermetallic Nanocrystals and Electrocatalytic Performance towards Oxygen Reduction Reaction

Lechao Peng [1], Lan Zhou [1], Wenjun Kang [1], Rui Li [1], Konggang Qu [1], Lei Wang [1], and Haibo Li [1,*]

1 Shandong Provincial Key Laboratory of Chemical Energy Storage and Novel Cell Technology, School of Chemistry and Chemical Engineering, Liaocheng University, Liaocheng 252059, PR China; lechaopeng@163.com (L.P.); lan_zhou_z@163.com (L.Z.); kangwenjun@lcu.edu.cn(W.K.); lirui@lcu.edu.cn (R.L.); qukonggang@lcu.edu.cn (K.Q.); wanglei@lcu.edu.cn (L.W.)

* Correspondence: haiboli@mail.ustc.edu.cn (H.L.)

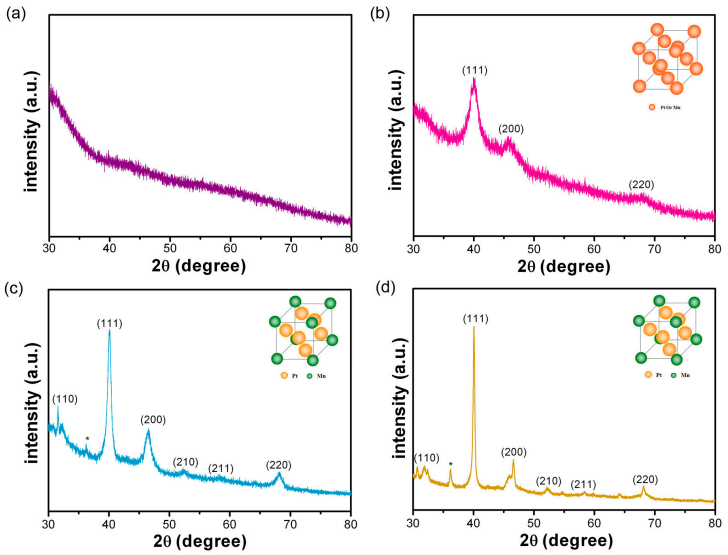

Figure S1. XRD patterns of (a) PtMn-650/CNFs, (b) PtMn-750/CNFs, (c) PtMn-850/CNFs, and (d) PtMn-1050/CNFs.

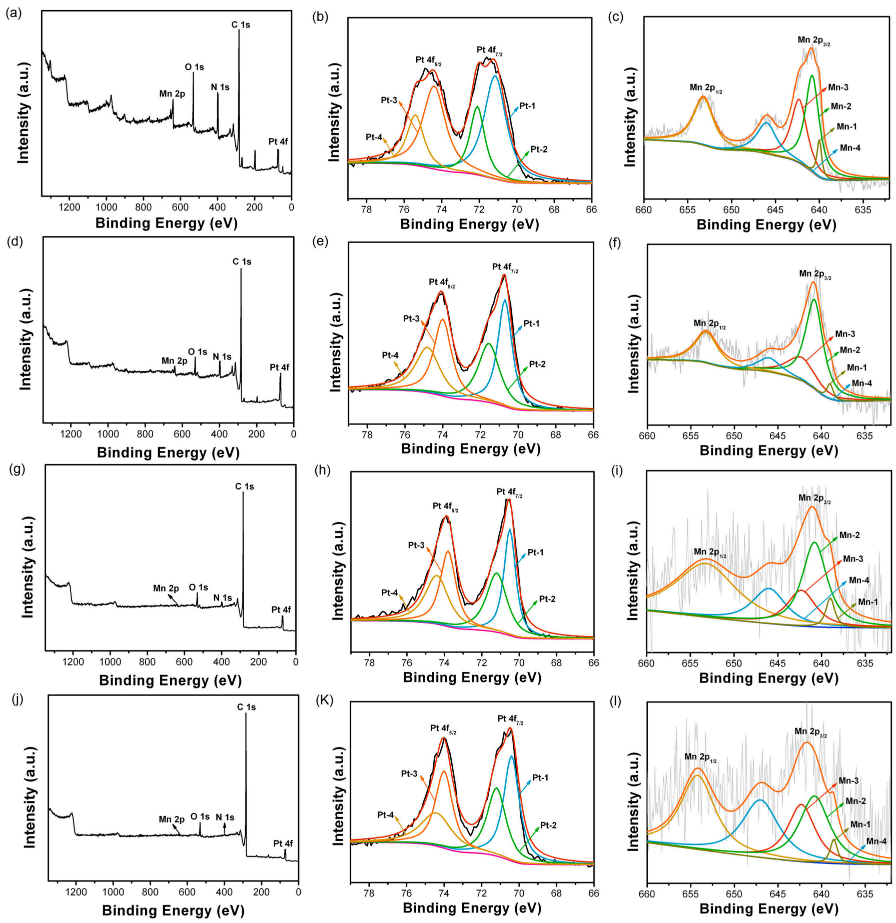

Figure S2. XPS survey spectra of (a) PtMn-650/CNFs, (d) PtMn-750/CNFs, (g) PtMn-850/CNFs, and (j) PtMn-1050/CNFs. Peak-fitting Pt 4f XPS spectra of (b) PtMn-650/CNFs, (e) PtMn-750/CNFs, (h) PtMn-850/CNFs, and (k) PtMn-1050/CNFs. Peak-fitting Mn $2p_{3/2}$ spectra of (c) PtMn-650/CNFs, (f) PtMn-750/CNFs, (i) PtMn-850/CNFs, and (l) PtMn-1050/CNFs.

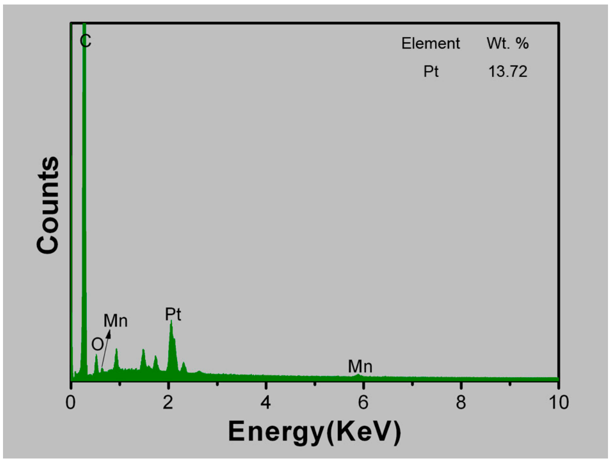

Figure S3. EDS of PtMn-950/CNFs.

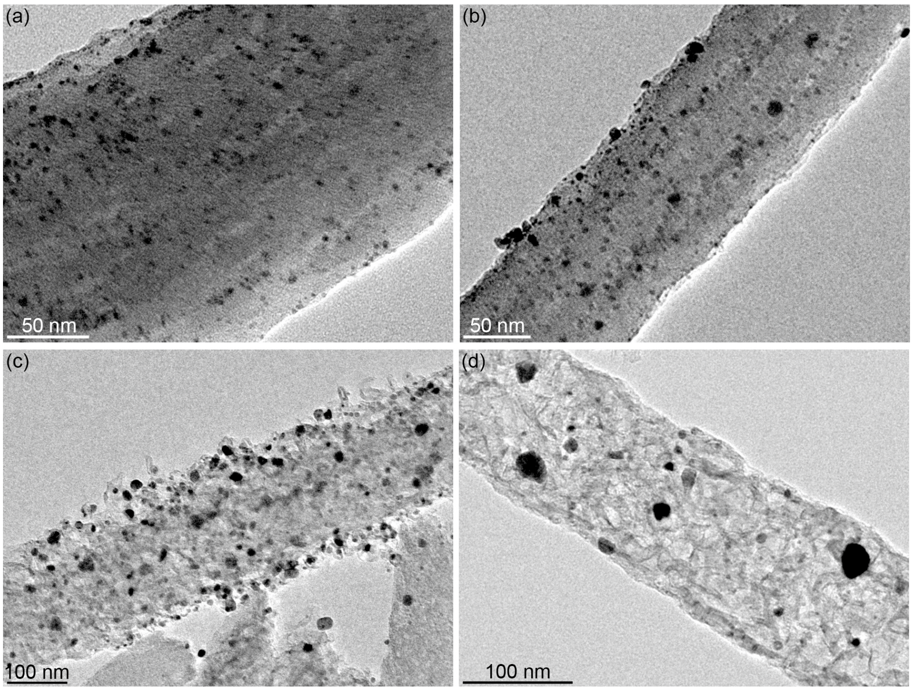

Figure S4. TEM images of (a) PtMn-650/CNFs, (b) PtMn-750/CNFs, (c) PtMn-850/CNFs, and (d) PtMn-1050/CNFs.

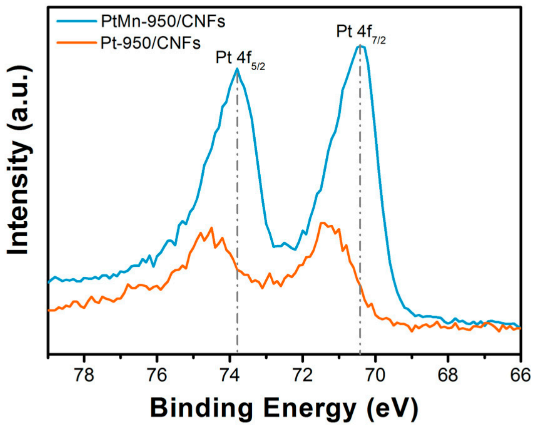

Figure S5. Pt 4f XPS spectra of PtMn-950/CNFs and Pt-950/CNFs.

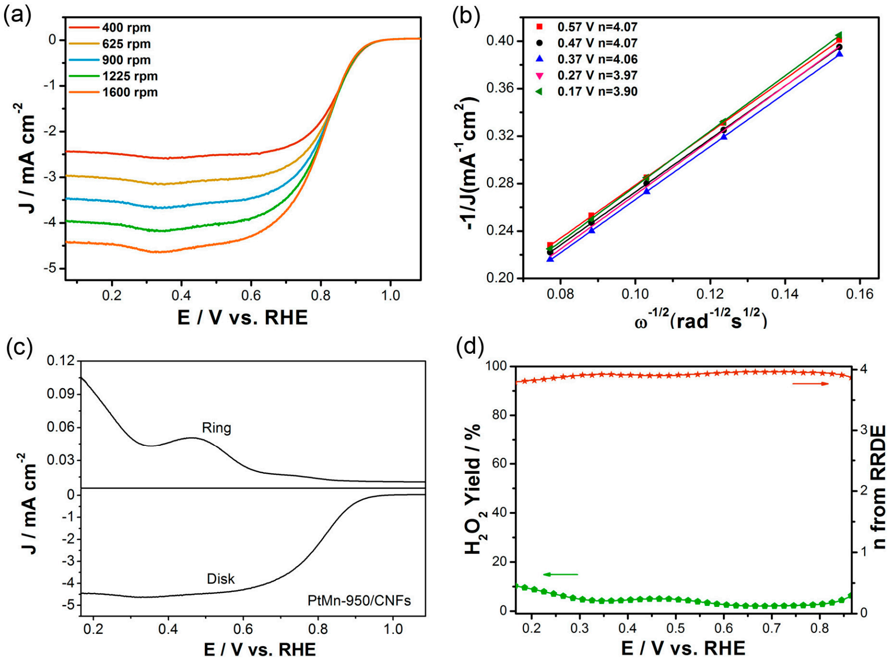

Figure S6. Electrochemical characterization of PtMn-950/CNFs after ADTs (5000 cycles): (a) ORR polarization curves at different rotation rates in O_2-saturated 0.10 M KOH; (b) Koutecky–Levich plots at different potentials; (c) ORR polarization curves recorded on the RRDE in O_2-saturated 0.10 M KOH solution at 1600 rpm; (d) the calculated H_2O_2 yield and electron transfer numbers (n).

Table S1. The calculation of the kinetic current densities at 0.832 V (vs. RHE)

Sample	PtMn-650/CNFs	PtMn-750/CNFs	PtMn-850/CNFs	PtMn-950/CNFs	PtMn-1050/CNFs
J / mA cm^{-2}	0.063	0.485	0.675	1.925	0.718
$1/J$ / mA^{-1} cm^{2}	15.80	2.06	1.48	0.52	1.39
J_L / mA cm^{-2}	2.19	2.65	2.64	4.31	3.20
$1/J_L$ / mA^{-1} cm^{2}	0.458	0.377	0.378	0.232	0.313
J_K^* / mA cm^{-2}	0.07	0.59	0.91	3.48	0.93

$$*JK = 1/(1/J - 1/JL)$$
